# Effect of Different Vitamin D Levels on Cognitive Function in Aged Mice After Sevoflurane Anesthesia

**DOI:** 10.3389/fnagi.2022.940106

**Published:** 2022-06-10

**Authors:** Jialei Zhang, Xiaoling Zhang, Yongyan Yang, Jun Zhao, Wenqing Hu, Yonghao Yu

**Affiliations:** ^1^Department of Anesthesiology, Tianjin Medical University General Hospital, Tianjin, China; ^2^Department of Anesthesiology, Changzhi People’s Hospital Affiliated to Changzhi Medical College, Changzhi, China; ^3^Department of Oncology, Changzhi People’s Hospital Affiliated to Changzhi Medical College, Changzhi, China; ^4^Department of Gastrointestinal Surgery, Changzhi People’s Hospital Affiliated to Changzhi Medical College, Changzhi, China

**Keywords:** vitamin D, sevoflurane, aged, POCD, inflammatory, cholinergic system

## Abstract

Although the biological relationship between vitamin D (VD) deficiency and cognitive function has been recognized by many scholars, the theoretical mechanisms involved are still not well-understood. In this study, we demonstrated the role of VD in alleviating the cognitive dysfunction in aged mice caused by sevoflurane anesthesia. Forty female C57BL/6 mice aged 12 months were selected for the experiment. VD (-) and VD (+) mouse models and sevoflurane anesthesia models were established. Mice were randomly divided into normal elderly group (NC group), normal aged mice + sevoflurane anesthesia treatment group (NS group), aged VD (-) mice + sevoflurane anesthesia treatment group [VD (-) group], and aged VD (+) + sevoflurane anesthesia treatment group [VD (+) group]. To compare the emergence time after sevoflurane anesthesia in aged mice with different levels of VD and to test the cognitive function of four groups through the water maze. Inflammatory factor expression and cholinergic activity in hippocampus tissue of all mice were measured at the end of behavioral tests. These data show that, low levels of VD aggravated the delayed emergence and cognitive dysfunction in aged mice caused by sevoflurane anesthesia, while higher levels of VD mitigated this impairment by enhancing cholinergic activity and reducing inflammatory factor expression in the hippocampus.

## Introduction

The aging population has become a focus of society as a whole, and with the advent of an aging society and advances in surgical techniques and anesthesia management, more elderly patients are being treated surgically and the incidence of post-operative cognitive dysfunction (POCD) is increasing and gaining more attention. POCD is one of the common complications after surgery, which is mainly manifested by the degeneration of cognitive functions such as memory, attention and information processing speed after anesthesia and surgery (Vutskits and Xie, 2016). The occurrence of POCD may prolong a patient’s hospital stay, increase the risk of death, consume a large amount of medical resources, and impose a heavy economic and social burden on patients and their families. Studies have shown that (Monk et al., 2008), advanced age is an independent risk factor for the development of POCD, regardless of the type of surgery. Delayed emergence is also a common complication after general anesthesia in elderly patients, and anesthesia and surgery are currently considered to be the main causes of its occurrence, while advanced age is a clear risk factor, with the degree of this risk increasing with age (Dringenberg and Olmstead, 2003).

Vitamin D (VD) deficiency has become a public health problem that affects the whole world, especially in the elderly population, and it is becoming more and more serious (Kweder and Eidi, 2018). It has been shown that (Groves et al., 2014; Jiang et al., 2014; Shin et al., 2016), VD as a neuroprotective hormone, is able to cross the blood-brain barrier to exert central protective effects by regulating the expression of key enzymes of neurotransmitter anabolism in the brain and is involved in a variety of brain activities including neurotrophy, neuroimmune regulation, and neurotransmission, and its deficiency is associated with a variety of neuropsychiatric disorders such as Parkinson’s disease, schizophrenia, and depression (Stewart et al., 2010; DeLuca et al., 2013).

In recent years, many studies have investigated the correlation between VD deficiency and POCD in elderly patients, but the results have not been very clear. Previous clinical studies by our team have shown that (Zhang et al., 2022), low levels of serum VD correlate with POCD in elderly patients. To further investigate the relationship between VD levels and perioperative cognitive function, we constructed an animal model of VD deficiency and supplementation in aged mice, compared the emergence time and cognitive function of aged mice with different levels of VD after sevoflurane anesthesia treatment, and measured and analyzed the levels of ChAT, IL-1β and TNF-α in hippocampus to determine whether VD deficiency could cause delayed awakening and cognitive function impairment in aged mice after anesthesia and its possible mechanism, and whether VD supplementation could reverse this impairment.

## Materials and Methods

### Experimental Animals

Forty female C57BL/6 mice aged 12 to 14 months [SCXK (Beijing) Biotechnology Co., Ltd., License No. SCXK (Beijing) 2019-0010] were selected for the experiment. All animals were numbered and grouped using the random number method with SPSS statistical software. Mice were randomly divided into normal elderly group (NC group), normal aged mice + sevoflurane anesthesia treatment group (NS group), aged VD (-) mice + sevoflurane anesthesia treatment group [VD (-) group], and aged VD (+) + sevoflurane anesthesia treatment group [VD (+) group], with 10 mice in each group. VD (-) and VD (+) mouse models and sevoflurane anesthesia models were established.

Vitamin D (-) mouse model was established: the outside of the cage of aged mice was covered with a shading curtain, without ultraviolet light, and the ratio of day to night was 12:12, keep the environment dry and ventilated, and feed them with special feeds that do not contain VD. After two months (during the pre-experiment, blood was collected once a month, and compared with normal aged mice, it was finally found that there was a difference in the dark for two months, in the formal experiment, the mice were directly kept in the dark for two months), blood was collected from the inner canthal vein. The content of VD [25 (OH) D3] was measured, and the difference was statistically significant compared with the normal elderly group, which was regarded as successful modeling.

Establishment of VD (+) mouse model: According to the literature (Jiang et al., 2014), 100 ng/kg calcitriol (Roche, Germany) was administered to aged mice in the VD (+) group by gavage every morning. One month later (during the pre-experiment, blood was collected once a month, and compared with normal aged mice, it was finally found that there was a difference in gavage for 1 month, and in the formal test, the gavage was chosen for 1 month), blood was collected from the inner canthal vein. The content of VD [25 (OH) D3] was measured, and the difference was statistically significant compared with the normal elderly group, which was regarded as successful modeling.

The normal elderly group was fed with normal feed, tap water and clean-grade feeding.

Feeding is carried out according to the results of the pre-experiment. Model feeding was performed on the aged mice in the aged VD (-) group first, and after one month, the aged mice in the aged VD (+) group were modeled and fed to ensure that all experimental mice were kept in the same age group.

After the model of VD (+) and VD (-) was successfully established, treated with sevoflurane anesthesia with the NS group together. Three groups were exposed to 3% sevoflurane and 60% O_2_ daily for 2 h in a closed environment for 3 consecutive days, while mice in the NC group were exposed to the same closed environment and inhaled only 60% O2 daily for 2 h for 3 consecutive days.

After the anesthesia time was over, the mice were removed from the confined environment and the recovery time of the flip reflex was recorded. According to the literature (Rebuelto et al., 2004), the recovery time of the flip reflex was defined as the ability to flip the mice to the prone position twice in a row within 2 s after flipping them to the dorsal recumbent position, and this time was taken as the emergence time.

After the pre-paration of the anesthesia model, the water maze directional navigation experiment was performed at a fixed time every morning on the 3th to the 7th day, and the water maze space exploration experiment was performed on the morning of the 8th day. All feeding programs were performed normally during the behavioral testing.

The experimental scheme was approved by the Ethics Committee of the Animal Experiment Center of Tianjin Medical University.

### Water Maze Experiment

The water maze experiment consisted of two parts: a navigation experiment and a spatial exploration experiment. This experiment is mainly used to test the long-term spatial memory of mice. The water maze consisted of a cylindrical tank with a diameter of 120 cm and a depth of 50 cm containing a platform with a diameter of 10 cm. Four signs with different colors and shapes were placed in symmetrical positions on the wall of the cylinder to facilitate learning and memory in the mice. An infrared monitoring device was installed at a central location over the water maze and was connected to external devices. System software (Xinxin, Shanghai) was used to track the movements of the mice. Before the experiment, the tank was filled with water to 1 cm above the platform, and titanium dioxide water color pigment was added to the water and mixed well such that the platform was not visible. At the same time, heating equipment was used to maintain the water temperature at 21°C. Under the monitoring equipment, the tank was divided into four quadrants I, II, III, and IV—and the platform was placed in the first quadrant (target quadrant). The experiment consisted of two parts: a positioning navigation experiment and a spatial exploration experiment. From the first to the fourth day of the experiment, the mice were placed into the water from the midpoints of the four quadrants, facing the wall of the cylinder. The time to find the platform was recorded, while swimming (latency time) followed by staying on the platform for 5 s. If the mouse could not find the platform within 120 s, it was artificially induced to find the platform and allowed to stay on it for 20 to 30 s; in this case, the latency time was recorded as 120 s. Each mouse was placed into the water at an interval of 20 min. On the fifth day of the experiment, the platform was removed, and the mice were placed in the water facing the wall at the center point of the quadrant opposite to the target quadrant (quadrant III). The proportion of time spent swimming in the target quadrant and the number of platform crossings were counted and recorded.

### Specimen Collection and Testing

The contents of VD in blood, ChAT,IL-1β and TNF-α in hippocampus were detected by ELISA.

After the model was successfully established, 0.1 ml of blood was collected from the medial canthal vein. After standing for 30 min, the supernatant serum was collected to detect the content of VD, which centrifuge at 4°C at 3000 r/min for 20 min. After all behavioral tests were completed, 0.5 ml of blood was collected by removing the eyeballs, and after standing for 30 min, centrifuged at 3,000 r/min at 4°C for 20 min, and the supernatant serum was collected to detect the content of VD. Mice were killed by dislocation method, hippocampal tissue was separated on ice, residual blood was washed with prefabricated PBS (0.01 mmol/L, pH = 7.40), weighed, cut into pieces, mixed with PBS solution, and ground on ice to a homogenized state, stand for 30 min, centrifuge at 5,000 r/min at 4°C for 10 min, take the supernatant. The contents of ChAT,IL-1β and TNF-α in hippocampus were determined by Elx800 microplate reader (Bio-Tek, United States).

### Statistical Analysis

IBM SPSS Statistics 26.0, which is produced by SPSS, Inc., was used for data analysis. To compare data within groups, the *t*-test was utilized. To compare data between groups, one-way ANOVA and two-way ANOVA were utilized. The water maze test data were analyzed using repeated measures ANOVA. At the same time, Pearson correlation analysis was performed on the VD value and inflammatory factors in mice to determine the relationship between VD content and the expression of inflammatory factors. *P* < 0.05 means the difference is statistically significant.

## Results

### Model Establishment

After aged mice in the VD (-) group were kept away from light and fed a VD-deficient diet for 2 months and those in the VD (+) group were administered calcitriol *via* gavage for 1 month, blood from the inner canthus vein was collected from NC group, NS group, and mice in the VD (+) and VD (-) groups. ELISA method was used to test the VD level of normal aged mice compared with VD (+) and VD (-) groups, and if the difference was statistically significant, the model was considered successful.

**FIGURE 1:**
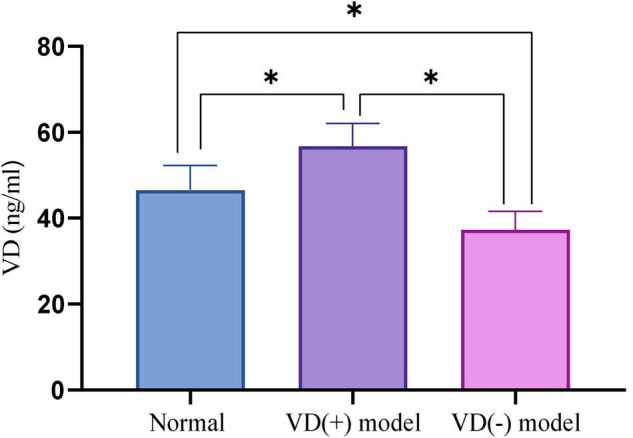
Comparison of VD content after successful modeling, *P < 0.05.

The results show that, VD (+) group (58.21 ± 5.62) has a VD concentration is higher than the NC group (45.30 ± 5.02) (*P* < 0.001), and the concentration of VD in VD (-) group (33.69 ± 5.33) is lower than NC group (*P* < 0.001). The data indicated that VD (+) and VD (-) models were established successfully (Figure 1).

### Comparison of Emergence Time Among the Anesthesia Treatment Group

**FIGURE 2:**
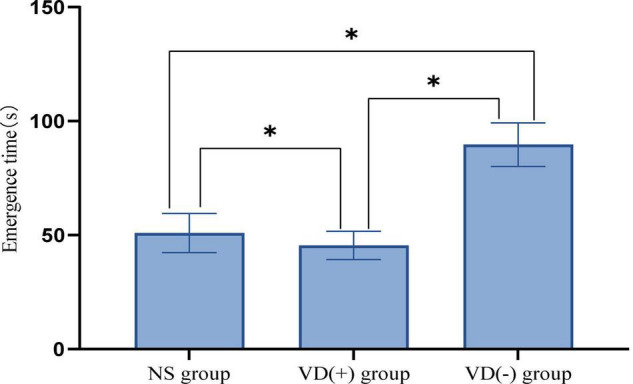
Comparison of emergence time among the anesthesia treatment group, *P < 0.05.

The experimental results showed that the emergence time of VD (-) aged mice was significantly longer than that of NS group (*P* < 0.001) and VD (+) group (*P* < 0.001), while the emergence time of aged VD (+) mice was significantly lower than that of NS group (*P* = 0.001) (Figure 2).

### Water Maze Test Results

In the water maze experiment, we conducted statistical analysis of each swimming escape latency time (positioning navigation experiment), the number of platform crossings (spatial exploration experiment) and the proportion of time spent in the target quadrant (spatial exploration experiment).

#### Comparison of Positioning Navigation Experiment (Escape Latency Time)

**FIGURE 3:**
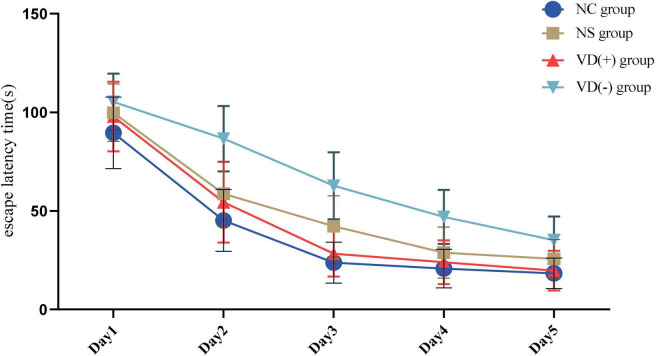
Comparison of the results of the positioning navigation experiment between groups.

Statistical results showed that the duration of escape latency time was significantly longer in the NS and VD (-) groups compared with the NC group (*P* < 0.05), while the VD (+) group was longer than the NC group on days 1 and 2 (*P* < 0.05), and no significant difference was seen between days 3 and 5 and the NC group (*P* > 0.05) (Figure 3).

Meanwhile, we conducted a one-way ANOVA on the anesthesia-treated NS, VD (+), and VD (-) groups (Table 1).

**TABLE 1:** One-way ANOVA for NS group, VD (+) group and VD (-) group.

	Day 1	Day 2	Day 3	Day 4	Day 5
NS group	99.79 ± 14.57	58.72 ± 16.47	42.17 ± 15.52	28.88 ± 12.95	25.64 ± 9.81
VD (+) group	97.87 ± 17.59	54.38 ± 20.43	28.23 ± 11.52	23.97 ± 11.11	19.75 ± 10.06
VD (-) group	105.35 + 14.26	86.63 ± 16.61	62.78 ± 16.98	46.93 ± 13.67	35.17 ± 11.98
F	2.496	38.096	54.757	36.655	21.277
*P*	0.087	<0.001	<0.001	<0.001	<0.001

#### Comparison of Spatial Exploration Experiment (the Number of Platform Crossings and Target Quadrant Time Percentage)

When comparing the number of platform crossings between groups, there was an overall decrease in the data for the NS, VD (+) and VD (-) groups after sevoflurane anesthesia treatment compared to the NC group. There was no statistical difference between the NC group and the VD (+) group (*P* = 0.482), but both groups were higher and statistically significant than the NS group (*P* = 0.003, = 0.019) and the VD (-) group (*P* < 0.001, < 0.001), while there was also a significant difference between the NS group and the VD (-) group (*P* = 0.008).

**FIGURE 4:**
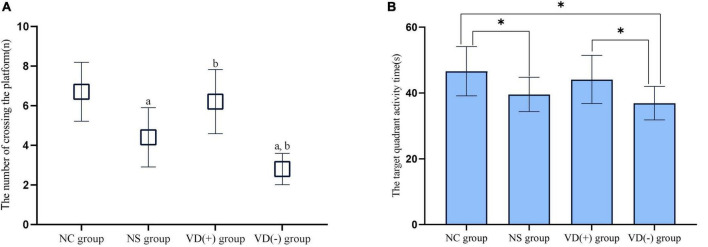
Comparison of experimental results of spatial exploration. **(A)** Comparison of the times of crossing the platform: comparison with NC group: *^a^P* < 0.05; comparison with NS group: *^b^P* < 0.05. **(B)** Comparison of target quadrant activity time ratio: comparison between groups: *P < 0.05.

Compared with the NC group, the target quadrant activity time of aged mice in the NS, VD (+) and VD (-) groups were significantly decreased. Similar to the results of the crossing platform, there was no statistical difference between the NC group and the VD (+) group (*P* = 0.456), also, no significant difference was seen between the NS and the VD (+) group (*P* = 0.129), but there was a significant difference between the NC and NS groups (*P* = 0.025). The target quadrant dwell time was shortest in the aged mice of VD (-) group, much lower than that in the NC group (*P* = 0.003) and the VD (+) group (*P* = 0.020), but there was no statistical difference than the NS group (*P* = 0.262) (Figure 4).

### Choline Acetyltransferase Vitality Results

#### Results of Choline Acetyltransferase Vitality Changes in the Hippocampus

**FIGURE 5:**
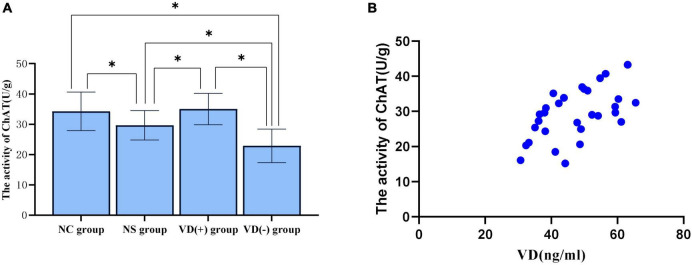
Comparison of ChAT activity in hippocampal region between groups and correlation analysis between VD level and ChAT activity. **(A)** Comparison of the vitality of ChAT between the groups, *P < 0.05. **(B)** Scatter plot of correlation analysis between VD and ChAT activity.

In this study, ChAT vitality was chosen to represent the altered cholinergic system in hippocampal tissue. The ELISA results showed that ChAT viability tended to decrease in NS, VD (+) and VD (-) group compared with NC group, and no significant abnormalities were seen between the NC and VD (+) groups (*P* = 0.470), but both were higher than the NS (*P* = 0.010, = 0.029) and VD (-) groups (*P* < 0.001, < 0.001), while the NS group was higher than the VD (-) group (*P* = 0.009) (Figure 5A).

#### Correlation Analysis Between Different Vitamin D Levels and the Vitality of Choline Acetyltransferase

Correlation analysis of VD levels and ChAT viability in three groups [NS group, VD (+) group and VD (-) group] of aged mice treated with sevoflurane anesthesia showed, between VD level and ChAT, the Pearson correlation coefficient was 0.554 (*P* = 0.002), that is, VD levels are positively correlated with ChAT activity in the hippocampus (Figure 5B).

### Inflammatory Markers Results

#### Expression of Inflammatory Factors in the Hippocampus

The results showed that the expression of TNF-α was significantly higher in the NS group, VD (+) group and VD (-) group compared to the NC group, but only the comparison of the VD (-) and NC groups showed significant differences (*P* < 0.001), there was no significant difference between the NC group and the NS group (*P* = 0.072), and with the VD (+) group (*P* = 0.794). A comparison of the NS, VD (+) and VD (-) groups treated with sevoflurane anesthesia showed that the TNF expression in the VD (-) group was much higher than that in the NS (*P* = 0.023) and VD (+) groups (*P* < 0.001), and also the difference between the NS and VD (+) groups was statistically significant (*P* = 0.030).

**FIGURE 6:**
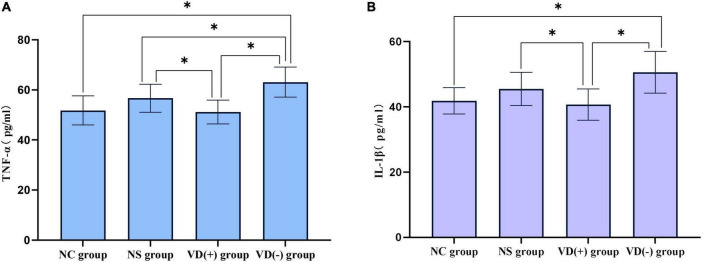
Comparison of inflammatory factors between the control group and the anesthesia treatment group. **(A)** Comparison of TNF-α: between groups, *P < 0.05. **(B)** IL-1β comparison: comparison between groups, *P < 0.05.

Analysis of IL-1β data showed that when comparing between groups, similar to TNF-α expression, the expression of IL-1β was significantly higher in the NS group, VD (+) group and VD (-) group compared to the NC group, but only the comparison of the VD (-) and NC groups showed significant differences (*P* = 0.002), there was no significant difference between the NC group and the NS group (*P* = 0.092), and with the VD (+) group (*P* = 0.568). Similarly, a comparison of the NS, VD (+) and VD (-) groups treated with sevoflurane anesthesia showed that the VD (+) group showed much lower IL expression than the NS (*P* = 0.043) and VD (-) groups (*P* = 0.001), while no significant difference was shown between the NS and VD (-) groups (*P* = 0.065) (Figure 6).

#### Correlation Analysis Between Different Vitamin D Levels and the Expression of Inflammatory Factors

**FIGURE 7:**
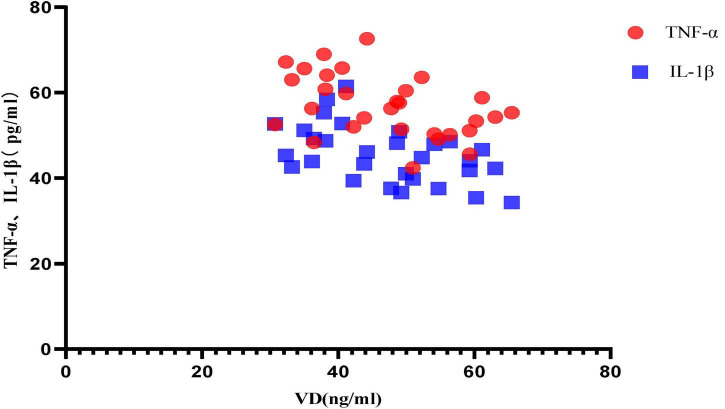
Scatter plot of correlation analysis between VD and inflammatory factors.

Correlation analysis between VD level and inflammatory factor expression in aged mice in anesthesia treatment group [NS group, VD (+) group and VD (-) group] showed that: between VD level and TNF-α, the Pearson correlation coefficient was −0.453 (*P* = 0.012), that is, VD was negatively correlated with TNF-α expression; between VD level and IL-1β, the Pearson correlation coefficient was −0.516 (*P* = 0.003), that is, VD was negatively correlated with IL-1β expression. VD is more strongly correlated with IL-1β (Figure 7).

## Discussion

With the accelerated aging of the global population, the proportion of elderly patients undergoing various types of anesthesia is increasing year by year due to the need for medical treatment or physical examination. It has been reported that about one-fourth of elderly patients undergoing major surgery have significant cognitive decline, and nearly half of them may develop permanent cognitive impairment (Kotekar et al., 2018). Meanwhile, Research studies have shown that VD deficiency is very common in the elderly, with more than half of them having this problem (Anthony et al., 2007). Although the biological relationship between VD deficiency and cognitive function has been recognized by many scholars, the relevant theoretical mechanisms are still unclear, and most of the current studies focus on clinical correlation studies, lacking in-depth studies on the intrinsic mechanisms.

In this study, we investigated the possible mechanisms by which VD protects brain function in aged mice by establishing both VD deficiency and VD supplementation models and by comparing behavioral changes in aged mice with different VD levels after sevoflurane anesthesia, as well as changes in the expression of inflammatory factors and ChAT activity in the hippocampal region of aged mice in each group. Research shows that reducing VD levels in adult mice below the VD-deficient state (12 ng/ml) (Holick et al., 2011) can lead to severe abnormalities in calcium and phosphorus metabolism, as well as limb weakness, slow movement, and significantly reduced mobility (Chang et al., 2011), which would affect the experimental results of this study. Therefore, we considered the modeling successful when an aged VD (-) model was established with lower VD content than normal aged mice and the difference was statistically significant. Similarly, the VD (+) model was successfully established when the VD content of aged VD (+) mice was higher than that of normal aged mice and the difference was statistically significant. Animal studies have shown that female mice are more prone to cognitive dysfunction than male mice (Zhang et al., 2017). Therefore, female mice were selected in this study to improve the detection rate of neurocognitive disorders after anesthesia.

Delayed emergence refers to the state that 2 h after the cessation of general anesthesia administration, excluding cerebrovascular accidents, consciousness does not return and cannot respond to speech or stimulation with thought, which is one of the common complications of general anesthesia (Tzabazis et al., 2015). It has been shown that advanced age has become an independent influencing factor for delayed emergence (Tsai et al., 2011). In this study, we performed statistical analysis on the emergence time of aged mice with different levels of VD after sevoflurane anesthesia, and the results showed that the emergence time of the VD (+) group was significantly better than that of the NS and VD (-) groups, and the VD (-) group was the longest, suggesting that VD can play a certain role in promoting emergence after sevoflurane anesthesia.

Morris water maze test is divided into positioning navigation test and space exploration test. The former reflects the spatial learning ability of animals, and the latter reflects the spatial memory ability of animals. It is a classic method for evaluating long-term spatial learning and memory in rodents (Tian et al., 2019). The results of the water maze spatial exploration experiment showed that, after sevoflurane anesthesia treatment, the number of platform crossing was significantly decreased in the NS group compared to the NC group, demonstrating that sevoflurane anesthesia caused some impairment of cognitive function in aged mice, which is consistent with previous findings (Fei et al., 2020; Wang et al., 2021). And when comparing aged mice with different VD levels after sevoflurane anesthesia, it was found that VD (+) aged mice were significantly better than the NS and VD (-) groups, demonstrating that high serum levels of VD could reduce the cognitive impairment caused by sevoflurane. In the water maze positioning navigation test, the VD (+) group showed differences with the NC group only in the first two days, but in the third to fifth days, there was no significant difference between the two groups, and it was significantly lower than the NS and VD (-) groups, which showed that the elderly mice with high VD levels had better learning and memory abilities. In contrast, the VD (-) aged mice performed the worst in both the localized navigation test and the spatial exploration test, confirming that low levels of VD may aggravate the cognitive dysfunction caused by sevoflurane anesthesia.

In recent years, some scholars have proposed the “central cholinergic hypothesis” related to the occurrence of POCD (Urits et al., 2019). The central cholinergic system is an important neurotransmitter system involved in a variety of body behaviors, plays an important role in the regulation of arousal, attention, and related learning functions (Deibel et al., 2016; Hampel et al., 2018). As the most important neurotransmitter in the central cholinergic system, Ach plays an important role in learning, memory, and regulation of the sleep-wake cycle, and when its synthesis and metabolism are affected, it causes impairment of the cholinergic system, thus affecting cognitive function and sleep-wake transition (Mohr et al., 2015; Xiong et al., 2019). However, Ach is extremely unstable and easily hydrolyzed, so difficult to accurately determine its content. ChAT is a specific enzyme for Ach synthesis in cholinergic neurons and is consistent with the distribution of Ach in the Central Nervous System (CNS), so it is often used as a marker of cholinergic neurons or as an indirect response to Ach content (Luo et al., 2009).

The results of this study showed that sevoflurane anesthesia caused a decrease in ChAT activity in the hippocampal region of aged mice compared with normal aged mice, which is consistent with the results of previous studies (Yang et al., 2019b). And when comparing different levels of VD aged mice after sevoflurane anesthesia, it was found that ChAT vitality in the VD (+) group was significantly higher than that in the NS group, and ChAT vitality in the VD (-) group was the lowest, meanwhile, correlation analysis between VD levels and ChAT vitality after anesthesia revealed that there was a positive correlation between them, i.e., the higher the VD, the better the ChAT vitality, while the VD decreased, the ChAT vitality declined.

Previous clinical and animal studies have found that sevoflurane has some neurotoxicity and can cause cognitive decline by mediating the inflammatory response (Fan et al., 2018; Yin et al., 2019). Therefore, we analyzed the expression of inflammatory factors in hippocampal tissue. The results showed that there was no significant difference in the expression of TNF-α and IL-1β in the hippocampus of normal aged mice without anesthetic treatment and aged mice treated with sevoflurane, but why are there behavioral differences between the two? It is speculated that this may be due to the fact that the specimens were collected on the 8th day after anesthesia treatment and missed the acute phase of inflammatory factor expression, so the difference in inflammatory factor expression could not be detected, at the same time, sevoflurane anesthesia treatment can lead to the destruction of the cytoskeleton and synaptic structure of neurons in the hippocampus, and this change is relatively persistent (Xiao et al., 2016; Yang et al., 2019a), so resulting in this phenomenon seen in this study. However, when comparing aged mice with different levels of VD after sevoflurane anesthesia treatment, we found significant differences. It was found that the expression of inflammatory factors in VD (+) aged mice was significantly lower than that in NS aged mice and VD (-) aged mice, and the expression of inflammatory factors in VD (-) aged mice was significantly higher than that in NS aged mice. Similarly, we conducted a correlation analysis between the level of VD and the expression of inflammatory factors, and the results showed that the level of VD was negatively correlated with the expression of TNF-α and IL-1β, that is, the higher the level of VD, the lower the expression of inflammatory factors, and the lower the level of VD, the higher the expression of inflammatory factors.

*In vivo* and *in vitro* studies have shown that (Zhao et al., 2019; Yu, 2021), sevoflurane anesthetic treatment can lead to reduced ChAT activity in the hippocampus of aged mice, which in turn leads to cognitive dysfunction in aged mice, presumably due to reduced ChAT activity, resulting in reduced Ach synthesis, impaired central cholinergic system, and inhibition of cholinergic anti-inflammatory pathways, along with increased expression of inflammatory factors in the brain stimulated by sevoflurane (Yang et al., 2019a; Yin et al., 2019), ultimately leading to POCD, which is consistent with the results of the present study. The results of the present study showed that VD levels in aged mice were positively correlated with ChAT activity in the hippocampal region and, at the same time, negatively correlated with inflammatory factor expression in the hippocampal region. Therefore, we hypothesized that higher VD levels could enhance ChAT activity, activate the cholinergic anti-inflammatory system, reduce the inflammatory response in the brain, and thus reduce the occurrence of POCD. However, the mechanism by which VD enhances ChAT activity is unclear and needs to be further investigated.

A large body of evidence suggests that VD can exert potential biological functions in brain tissue and protect the nervous system, but the exact mechanism of action is unclear. It was found that pretreatment with VD ameliorated 6-hydroxydopamine-induced motor dysfunction and neuronal toxicity and greatly reduced the amount of neuronal apoptosis (Wang et al., 2001). In addition, similar to conventional antioxidants, VD inhibits the production of nitric oxide synthase (NOS), an enzyme whose expression is significantly elevated in CNS neurons and non-neurons in response to physical injury or disease, particularly in ischemic events, Alzheimer’s disease, and Parkinson’s disease (Chang et al., 2004). VD also increases γ-glutamyl transpeptidase levels by activating intrinsic antioxidant pathways (Garcion et al., 1999), thereby increasing glutathione levels and protecting the integrity of oligodendrocytes and neurotransmission pathways. Recent studies have shown that VD can elevate ACH levels and attenuate the resulting cognitive impairment by inhibiting the expression of neuroinflammatory factors including IL-1β, BDNF, and NF-κB caused by a high-fat diet (Farhangi et al., 2017).

In conclusion, our data show that sevoflurane-induced cognitive dysfunction in aged mice is associated with decreased cholinergic system viability and increased inflammatory factor expression in the hippocampal region, and that decreased VD levels *in vivo* can aggravate this impairment, which can be alleviated by pre-supplementation with VD, this may be related to the fact that VD increases ChAT activity, thereby activating central cholinergic anti-inflammatory pathways and inhibit the inflammatory factor expression in the brain. This finding may serve as a new therapeutic measure to clinically mitigate cognitive dysfunction in elderly patients after perioperative sevoflurane anesthesia, but until then, it still needs to be confirmed by numerous clinical trials, especially to qualify the optimal daily dose of VD.

## Data Availability Statement

The original contributions presented in the study are included in the article/supplementary material, further inquiries can be directed to the corresponding author.

## Ethics Statement

The experimental scheme was approved by the Ethics Committee of the Animal Experiment Center of Tianjin Medical University.

## Author Contributions

JiZ and YYu designed the study and wrote the manuscript. JiZ, XZ, YYa, and JuZ performed the experiments. JiZ, XZ, and WH analyzed the data. All authors contributed to the article and approved the submitted version.

## Conflict of Interest

The authors declare that the research was conducted in the absence of any commercial or financial relationships that could be construed as a potential conflict of interest.

## Publisher’s Note

All claims expressed in this article are solely those of the authors and do not necessarily represent those of their affiliated organizations, or those of the publisher, the editors and the reviewers. Any product that may be evaluated in this article, or claim that may be made by its manufacturer, is not guaranteed or endorsed by the publisher.
